# A Comparison of β-Carotene, Phytoene and Amino Acids Production in *Dunaliella salina* DF 15 (CCAP 19/41) and *Dunaliella salina* CCAP 19/30 Using Different Light Wavelengths

**DOI:** 10.3390/foods10112824

**Published:** 2021-11-16

**Authors:** Yixing Sui, Laura Mazzucchi, Parag Acharya, Yanan Xu, Geraint Morgan, Patricia J. Harvey

**Affiliations:** 1 Aquatic Biotechnology and Biology, Natural Resources Institute, Faculty of Engineering and Science, University of Greenwich, Central Avenue, Chatham Maritime, Kent ME4 4TB, UK; y.sui@greenwich.ac.uk (Y.S.); l.mazzucchi@greenwich.ac.uk (L.M.); P.Acharya@greenwich.ac.uk (P.A.); Y.Xu@greenwich.ac.uk (Y.X.); 2 School of Physical Sciences, The Open University, Milton Keynes MK7 6AA, UK; geraint.morgan@open.ac.uk

**Keywords:** microalgae, carotenoids, LED, novel food, halophile

## Abstract

Strains of *Dunaliella salina* microalgae are of considerable research and industrial interest because they hyper-accumulate β-carotene as well as produce high-quality protein. To explore the co-production of valuable compounds in *D. salina*, this study compared the production of β-carotene, phytoene and amino acids in two strains cultivated under white, red or blue light until no further nitrogen was available. *D. salina* DF15 (CCAP 19/41 (PLY DF15)) produced more than 12% β-carotene (ash-free dry weight (AFDW) basis), and red light triggered the production of *9-cis* β-carotene at a *9-cis*/*all-trans* β-carotene ratio of 1.5. Phytoene production was also evident in *D. salina* DF15 under all conditions, particularly under blue light. However, the profile of essential amino acids (EAAs) and calculation of the essential amino acid index (EAAI) was less than ideal in terms of protein quality, for both strains. Umami compounds, quantified as monosodium glutamate (MSG) equivalents, indicated a higher equivalent umami concentration (EUC) in *D. salina* DF15 under red light (3.2 g MSG/100 g AFDW) than in *D. salina* CCAP19/30. Overall, *D. salina* DF15 demonstrates valuable traits for further exploration and product optimisation.

## 1. Introduction

Many microalgal species have been studied for their suitability as sustainable sources of natural carotenoids for the Blue Bioeconomy. Carotenoids are the precursors of a range apocarotenoids of biological and commercial importance, such as the phytohormone abscisic acid, the visual and signalling molecules retinal and retinoic acid, and the aromatic volatile beta-ionone [1,2,3]. The global carotenoids market is expected to grow by 5% until 2031, with sales likely to surpass USD two billion (https://www.factmr.com/report/1196/carotenoids-market accessed on 3 November 2021). The market share of β-carotene exceeds USD 520 million, and there is a growing demand (at a rate of 6.5% market growth) for naturally sourced β-carotene owing to increased consumer inclination towards additive-free natural products in the healthcare and food sectors (https://www.gminsights.com/industry-analysis/beta-carotene-market accessed on 3 November 2021). *Dunaliella salina* is renowned for its unique β-carotene overproduction: under stress conditions, cells reach up to 10% of their dry weight in β-carotene [4]. This alga is one of the most used phototrophic species grown with (sun)light as the energy source at industrial scale. Two isomers of β-carotene, namely, *all-trans* and *9-cis* β-carotene, have been identified and quantified in *D. salina* as the major constituents [5]. Due to its higher antioxidant activity, *9-cis* β-carotene is of widespread industrial interest, and has been shown to hyper accumulate in *D. salina* DF15 (CCAP 19/41 (PLY DF15)) under red light [5]. *D. salina* DF15 has also been shown to produce valuable quantities of the colourless carotenoids phytoene and phytofluene in the presence of a cell division inhibitor under red light [6]. Nevertheless, stress conditions may hinder the growth and accumulation of other valuable compounds.

In recent years, the potential of *D. salina* to be used as a sustainable protein source has bloomed, even after the separation of carotenoids [7,8]. However, it has been reported that *D. salina* protein quantity and quality can be affected by various factors, including nutrient levels, light intensities and light wavelengths, among others [9,10,11]. As a result, the essential amino acid (EAA) content and essential amino acid index (EAAI) have also been reported with different values, indicating variable protein qualities [9,10,11]. In several studies, cultivation with either nitrogen, phosphorus or sulphur limitations each modified the amino acid profile and changed the EAAI in *D. salina* [9,12,13]. On the other hand, cultivation under white, red or blue light wavelengths, which were previously reported to alter β-carotene isomeric composition in *D. salina* DF15 when nitrogen was abundant [5,14], did not seem to change the EAA profiles [11]. This effect might be strain-dependent, because carotenoid profiles are differently affected depending on the nature of the *D. salina* strain used [14], rather than an effect of nitrogen abundance failing to discriminate differences between white, red or blue light on the EAA profile [11]. The potential of *D. salina* to co-produce high-quality protein and carotenoids has previously been demonstrated, where two-phase cultivation using nitrogen limitation and high light intensity was adopted [9]. In this study, β-carotene isomeric compositions with respect to *all-trans* and *9-cis* β-carotene, phytoene and amino acids production were investigated once nitrogen was depleted, in two *Dunaliella salina* strains: *D. salina* DF 15 (CCAP 19/41), which is known for β-carotene hyper-accumulation, and *D. salina* CCAP 19/30, which is a model green-phase fast-growing strain and very similar to a *Dunaliella tertiolecta* strain which does not accumulate β-carotene under stress [15].

## 2. Materials and Methods

### 2.1. Microalgal Strains and Cultivation Methods

*Dunaliella salina* PLY DF15 (CCAP 19/41) was obtained from the Marine Biological Association (MBA, Plymouth, UK and *Dunaliella salina* CCAP 19/30, from the Culture Collection of Algae and Protozoa at Scottish Marine Institute (CCAP, Oban, Scotland, UK). Modified Johnson’s Medium [16] with 0.1 g/L KNO_3_, 10 mM Tris buffer, 87.75 g/L (1.5 M) NaCl at pH 7.5 was used for the single-phase batch cultivation. Each *Dunaliella* strain was cultivated in Algem^®^ photobioreactors from Algenuity (Stewartby, Bedfordshire, UK) (https://www.algenuity.com/ accessed on 3 November 2021) at a continuous light intensity of 200 µmol photons m^−2^ s^−1^ using either white, blue or red light emitting diode (LED) light (Figure S1), resulting in a total of six treatments, namely, DF15_White, DF15_Red, DF15_Blue, CCAP 19/30_White, CCAP 19/30_Red and CCAP 19/30_Blue. Stock cultures were grown to mid-log phase and 1 mL stock culture was inoculated to 20 mL medium in each 50 mL Erlenmeyer flask to reach an optical density (OD) of around 0.02 at 740 nm. The temperature in the photobioreactors was controlled at 25 °C and the mixing was provided by 100 rpm orbital shaking. All treatments were performed in triplicates in 50 mL Erlenmeyer flasks with 20 mL culture.

### 2.2. Biomass, Carotenoids and Amino Acids Content Analysis

Cell growth was monitored by recording the value obtained for in vivo absorbance at 740 nm in OD units (OD740) every 2–3 days by taking 0.3 mL samples in a 96-well microplate for optical density readings in a microplate reader (Thermo Scientific Multiskan GO, Oxford, UK). The total microalgal biomass was harvested at the end of the experiment when the growth curve flattened to reach the stationary phase. The maximum specific growth rate was calculated between day 0 and day 14.

Glass fibre filters (Millipore, Ø 24 mm, pore size 0.7 µm) were used to gravimetrically determine the biomass ash-free dry weights (AFDW). Subsequently, 1.3 mL microalgal suspension was filtered through a pre-cleaned glass fibre filter and then dried at 105 °C overnight in an oven (Fistreem International Ltd., Cambridge, UK). After cooling down in a desiccator, weight A was recorded before the filter was transferred to a 550 °C muffle furnace (Vecstar Ltd., Chesterfield, UK). After 2 h in the furnace, the filter was cooled down in a desiccator again before weight B was recorded. The AFDW was determined following the equation:

(1) $$AFDW\;\left(g/L\right)=\frac{A-B}{V}$$

where *A* and *B* (mg) are the recorded weights, and *V* is the volume of filtered microalgal suspension (1.3 mL). The filtrate was collected for nitrate determination using a testing kit (Hach Lange LCK 339, Manchester, UK) and a spectrophotometer (Hach Lange DR3900, Manchester, UK).

Before determining the protein content of microalgal biomass, the samples were incubated for 1 h in deionised water at 40 °C in a thermoshaker (Grant Instruments Ltd., Shepreth, UK). A Thermo Scientific™ Pierce™ modified Lowry protein assay kit was used for protein determination.

Prior to amino acid analysis, microalgal suspension was pelletised by centrifugation at 5000× *g* for 5 min. The biomass pellet was then hydrolysed with 6 M HCl in a vacuum-sealed ampule glass tube for 24 h at 110 °C. The hydrolysate was used for sample preparation following the Phenomenex EZ:faast amino acid analysis kit [17] coupled with a gas chromatography system with a flame ionization detector (GC-FID) (Agilent 7890A, Stockport, UK). Bovine serum albumin (BSA) was used as control to calculate the amino acid recovery. The essential amino acid index (EAAI) was calculated following this equation:

(2) $$EAAI=\sqrt[{n}]{\frac{aa1}{AA1}\times \frac{aa2}{AA2}\times \ldots \ldots \times \frac{aan}{AAn}}$$

where *aan* and *AAn* are the EAA content (mg EAA g^−1^ protein) in the sample and referenced by Food and Agriculture Organization/World Health Organization (FAO/WHO), respectively. The protein quality was categorized as: “high” (EAAI > 0.95), “good” (0.86 < EAAI ≤ 0.95), “useful” (0.75 < EAAI ≤ 0.86), and “inadequate” (EAAI ≤ 0.75) [14].

The equivalent umami concentration (EUC) was used to evaluate the umami taste intensity by converting umami compounds into the concentration of monosodium glutamate (MSG), following this adapted equation from:

(3) $$Y=\sum a_{i}b_{i}$$

where *Y* is the EUC (mg MSG/100 g AFDW), *a_i_* is the concentration of glutamate and aspartate (g/100 g AFDW), and *b_i_* is the relative umami concentration of glutamate (1) and aspartate (0.077).

To determine carotenoids, microalgal biomass was harvested from 7 mL cultures from each treatment of both strains by centrifugation at 3000× *g* for 5 min and pigments were extracted using methanol (MeOH) and methyl tert-butyl ether (MTBE) as follows: 7 mL MeOH/MTBE (80/20) was added to the pellets, sonicated, and vortexed for 20 s, similarly to the procedure in [6]. The extracts were centrifuged at 3000× *g* for 5 min and the supernatant was filtered (0.20 µm filter) into amber high-performance liquid chromatography (HPLC) vials before HPLC analysis. 

High-performance liquid chromatography equipped with diode-array detection (HPLC-DAD; Agilent Technologies 1200 series, Agilent, Santa Clara, CA, USA), an online degasser, a quaternary pump system, and a YMC30 250 × 4.9 mm I.D S-5 µ column (YMC Europe GmbH, Dinslaken, Germany) was used to detect and quantify β-carotene and phytoene from *D. salina* carotenoid extracts. The column temperature was set at 20 °C, the isocratic solvent system was MeOH (A):MTBE (B) running at 80%:20% A:B and the flow rate was 1 mL/min at a pressure of 78 bar, as described previously [6]. The run time was 45 min. The detector operated in the wavelength range 210–663 nm. The β-carotene and phytoene standards were purchased from Sigma-Aldrich Inc. (Merck KGaA, Darmstadt, Germany) and LGC Limited (Teddington, UK), respectively. The standards were dissolved in Methanol:MTBE (80:20) and were identified based on the retention time parameters and absorption spectrum at 450 nm (for β-carotene) and 280 nm (for phytoene) to generate the standard curves for quantification.

### 2.3. Statistical Analysis

Triplicates were set up for each treatment in the experiment. The results are expressed as mean ± standard deviation in the figures and tables. For better readability, only mean values are stated in the text. IBM SPSS Statistics 26 was used to perform one-way ANOVA followed by post hoc Tukey’s test with a significant level of *p* < 0.05.

## 3. Results

### 3.1. Biomass Growth

**Figure 1:**
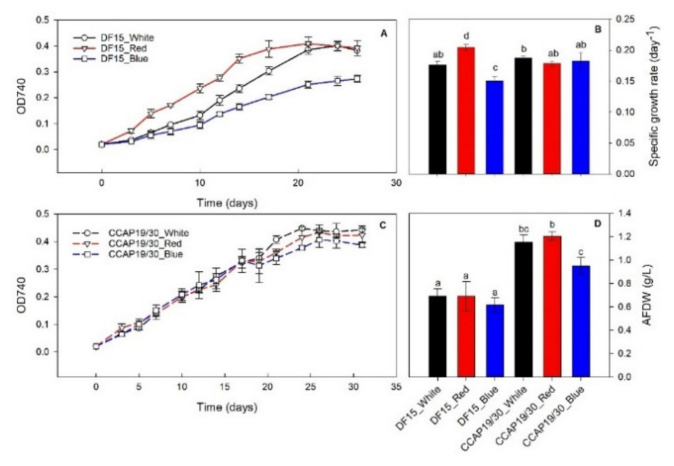
Growth curve at OD740 of *D. salina* DF15 (**A**), *D. salina* CCAP19/30 (**C**), their maximum specific growth rate and (**B**) and final biomass concentration (**D**). Cultivation was conducted at 25 °C, pH 7.5 and 100 rpm mixing using white, red and blue light at 200 µmol photons m^−2^ s^−1^. Data are expressed as the means ± standard deviation. The different letters indicate statistically different groups (significance level at *p*-value < 0.05).

Different growth profiles were obtained for *D. salina* DF15 and *D. salina* CCAP 19/30 during the course of their growth (Figure 1). For *D. salina* DF15 (Figure 1A,B), red and blue light resulted in the fastest and slowest growth, respectively, among the three light wavelengths tested, with significant differences (*p* < 0.05). The growth profiles of *D. salina* CCAP 19/30 were not affected by light wavelengths during the initial phase of growth compared to *D. salina* DF15. This can be seen by the insignificant differences in the maximum specific growth rates measured for CCAP 19/30 under either white, red or blue light (Figure 1B). Nevertheless, towards the end of the growth, the growth of CCAP 19/30 slowed down more under blue light than under white or red light. Similar to [15], at the end of the experiment, the amount of biomass that accumulated in cultures of *D. salina* CCAP 19/30 was significantly higher (0.95 to 1.21 g AFDW/L across three light wavelengths) compared with *D. salina* DF15, which reached between 0.62 and 0.69 g AFDW/L (Figure 1B). For both strains, biomass loss was evident under white, red or blue light towards the end of the experiment, but blue light led to impaired biomass growth throughout the growth period, which can be seen from the maximum specific growth rate and the final AFDW. Nitrate concentrations in the media from all treatments were below detection limits, indicating nitrogen starvation (data not shown).

### 3.2. Protein and Amino Acids

**Figure 2:**
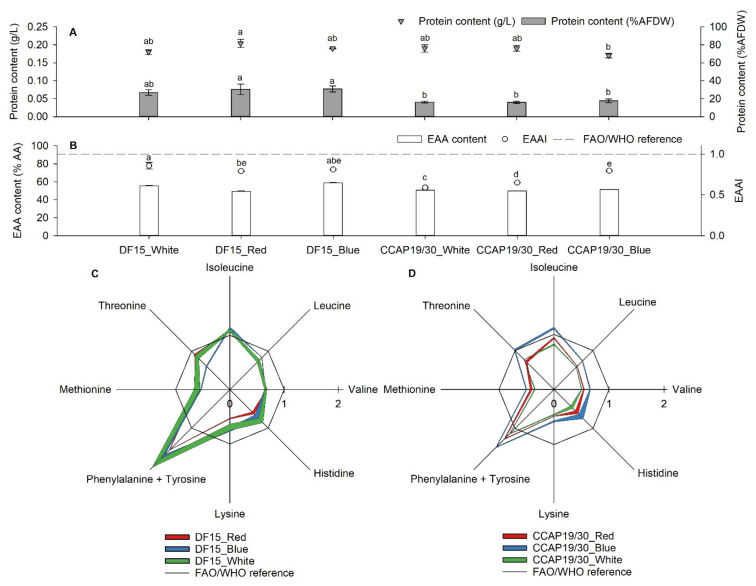
Protein content (**A**), EAA content, EAAI (**B**) and EAA profile of *D. salina* DF15 (**C**) and *D. salina* CCAP19/30 (**D**) at the end of the experiment. Cultivation was conducted at 25 °C, pH 7.5 and 100 rpm mixing using white, red and blue light at 200 µmol photons m^−2^ s^−1^. Data are expressed as the means ± standard deviation. The different letters indicate statistically different groups (significance level at *p*-value < 0.05).

Figure 2 shows the protein content, EAA content, EAAI, and individual EAA level of the two *D. salina* strains under three light wavelengths. Despite differences in biomass yield (Figure 1D), protein yields (g/L) in the microalgal suspension from all treatments of the two strains were quite similar (ranging from 0.17 to 0.2 g/L) apart from the differences between *D. salina* DF15 under red light (greatest) and *D. salina* CCAP 19/30 under blue light (least) (Figure 2A). The protein yields reflected significant differences (*p* < 0.05) in protein content (% AFDW) recorded for the strains under different light intensities (Figure 2A). *D. salina* DF15 under white, red and blue light resulted in 27%, 30% and 31% protein content (% AFDW), respectively, whereas *D. salina* CCAP 19/30 only showed 16%, 16% and 18% protein content for the corresponding light treatments. 

In terms of the protein quality, both *D. salina* strains presented similar EAA content, around 50% over total amino acid (AA) content (Figure 2B). Nevertheless, the EAAI values in *D. salina* DF15 under all light wavelengths were significantly higher than for *D. salina* CCAP 19/30, except for that under blue light (Figure 2B). White, red and blue light resulted in EAAI values of 0.86, 0.79 and 0.81 in *D. salina* DF15, respectively, which are all categorised as “useful” protein, compared to EAAI values of 0.59, 0.65 (“inadequate” protein) and 0.80 (“useful” protein) in *D. salina* CCAP 19/30 (Figure 2B). Overall, the protein quality of *D. salina* biomass achieved once nitrogen was fully depleted was neither “good” nor “high” in quality, which can also be seen by the individual EAA levels in reference to the FAO/WHO levels (Figure 2C and Table 1). Only a few amino acids, including isoleucine, phenylalanine + tyrosine and threonine, passed the recommended level set by the FAO/WHO, whereas all other amino acids were below the threshold. EAA concentration/g protein (Table 1) and patterns (Figure 2C) were similar among all light wavelengths in *D. salina* DF15, but for *D. salina* CCAP 19/30, blue light treatment gave higher EAA concentrations/g protein than for white and red light, and significantly higher EAAI values. The overall higher individual EAA concentrations/g protein resulted in significantly higher total EAA in *D. salina* DF15 than in *D. salina* CCAP 19/30 (*D. salina* CCAP 19/30 under blue light is an exception).

**Figure 3:**
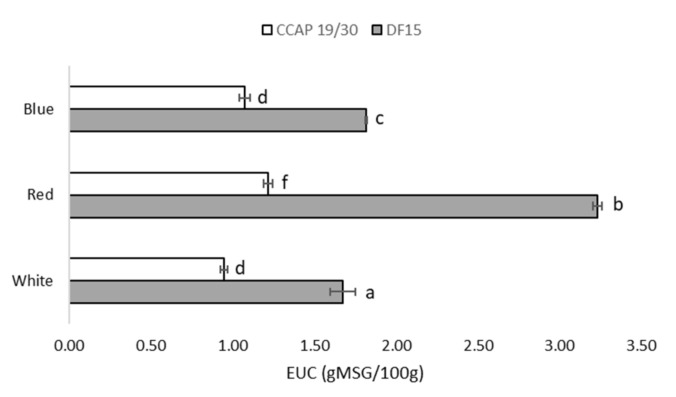
Equivalent umami concentration (EUC) of *D. salina* DF15 and *D. salina* CCAP19/30 at the end of the experiment. Cultivation was conducted at 25 °C, pH 7.5 and 100 rpm mixing using white, red and blue light at 200 µmol photons m^−2^ s^−1^. Data are expressed as the means ± standard deviation. The different letters indicate statistically different groups (significance level at *p*-value < 0.05).

Non-essential amino acids which contribute to “taste” characteristics were also quantified. As seen in Figure 3, the equivalent umami concentration (EUC) of *D. salina* DF15 surpassed the EUC of *D. salina* CCAP 19/30 under all light wavelengths, with significant differences. Red light resulted in the highest EUC in *D. salina* DF15, reaching 3.2 g MSG equivalents/100 g AFDW, compared to 1.67 and 1.82 g MSG equivalents/100 g AFDW under white and blue light, respectively. The same trend was present in *D. salina* CCAP 19/30, where red light resulted in the highest EUC compared to white or blue light. 

### 3.3. Carotenoids Production

**Figure 4:**
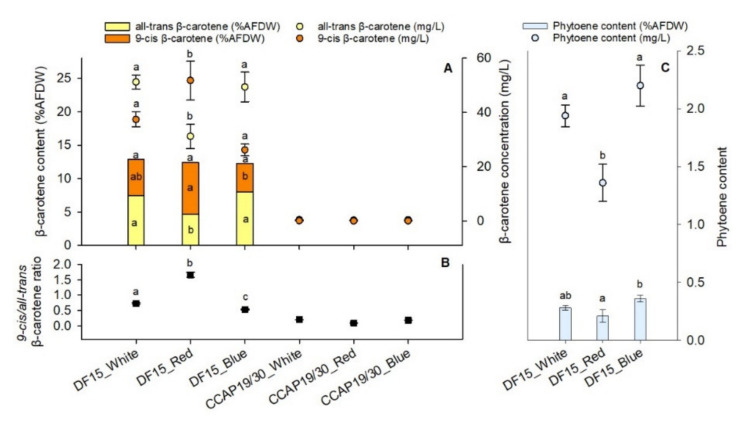
The *9-cis* and *all-trans* β-carotene contents (**A**), *9-cis*/*all-trans* β-carotene ratio (**B**) and phytoene content (**C**) of *D. salina* DF15 and *D. salina* CCAP19/30 at the end of the experiment. Cultivation was conducted at 25 °C, pH 7.5 and 100 rpm mixing using white, red and blue light at 200 µmol photons m^−2^ s^−1^. Data are expressed as the means ± standard deviation. Different letters indicate statistically different groups for *D. salina* DF15 (significance level at *p*-value < 0.05).

*D. salina* DF15 is a patented strain [18] which has the potential to produce a large quantity of carotenoids compared to other strains. As shown in Figure 4A, the total β-carotene contents (*all-trans-*plus *9-cis-*isomers) in *D. salina* DF15 under all light wavelengths were much higher than those in *D. salina* CCAP 19/30, which barely showed any carotenoid production (hence, their exclusion from the statistical analysis). The total β-carotene contents in *D. salina* DF15 suspension reached 89, 83 and 76 mg/L, accounting for 12.9%, 12.4% and 12.3% AFDW (without significant differences) using white, red and blue light, respectively. However, the fraction of *all-trans-* and *9-cis* β-carotene under red light showed a significant difference. Red light significantly enhanced the production of *9-cis* β-carotene in *D. salina* DF15 by 38%, from 37.4 mg/L to 51.8 mg/L, compared to white light. Blue light resulted in the significantly lowest production of *9-cis* β-carotene of only 26.2 mg/L. When looking at the ratio of *9-cis* to *all-trans* β-carotene, red light showed an increase of more than double compared to white light, and even more than a tripled increase compared to blue light, all with significant differences (Figure 4B).

Interestingly, another valuable carotenoid, phytoene, was detected in *D. salina* DF15; however, no phytoene was detected in *D. salina* CCAP 19/30 (Figure 4C). Differently from *9-cis* β-carotene, red light resulted in the lowest yield of phytoene at 1.4 mg/L (0.21% AFDW), whereas blue light exhibited the highest phytoene yield of 2.2 mg/L (0.36% AFDW). The differences between red and blue light in phytoene yield were also statistically different (Figure 4C).

## 4. Discussion

### 4.1. Protein and Amino Acid Dynamics

Microalgae present diverse protein quantities and qualities across species and strains, which depends on N assimilation coupled to the tricarboxylic acid (TCA) cycle; when nitrogen assimilation is impaired, the flow of carbon skeletons diminishes and may lead to a hyperoxidant state and cell death if oxygen reduction in respiration is reduced [19]. In most microalgae, the preferred form of N is ammonium, which has a lower metabolic cost to reduce to organic matter than the cost for other forms such as nitrate [20]. In *Dunaliella* cells, however, inorganic nitrogen in the form of nitrate NO_3_^−^, not ammonium, is the preferred form: these cells are bound by an outer cytoplasmic membrane to adjust their volume and shape rapidly in response to hypo- or hyper-osmotic changes in the external environment, and NH_4_^+^, in equilibrium with NH_3_ diffusing passively into the cells across the plasma membrane, depletes TCA cycle intermediates. This, in turn, disrupts the cellular respiration required for adenosine triphosphate (ATP) production and oxygen reduction to water and results in reduced biomass accumulation [21]. Similarly, when nitrogen becomes limiting, microalgal cells may activate nitrogen scavenging mechanisms to acquire, remobilise and redistribute intracellular nitrogen in order to maintain carbon flow and oxygen reduction, which can result in an increase in amino acids relative to total biomass [13,22,23,24,25]. Protein quality, measured in terms of EAA content and the EAAI, may improve as cells redirect their metabolism to increase the degradation of certain amino acids and repress the biosynthesis pathways of others; glutamate levels may decline along with the cellular amino acid pool, but stress-related amino acids such as proline, lysine and tyrosine may increase [22,23,24,25,26,27]. Both *D. salina* CCAP 19/18 and *D. salina* DF15 have been shown to support a high protein quality measured in terms of EAAI and EAA content under N-limiting conditions [9,11]. Protein quality can also depend on light quality: in *Nannochloropsis gaditana* under high-energy blue light, carbohydrate concentrations decreased compared to under red light, and the stress-related amino acid proline increased [28]. Similarly, all proline (data not shown), lysine and tyrosine levels in our results (Table 1) also indicated an increased trend for *D. salina* CCAP 19/30 under blue light compared to both red and white light. For *D. salina* DF15, the increase only occurred for lysine and tyrosine under blue light compared to red light, but not white light (Table 1). In *Spirulina* sp. LEB 18, low-energy red light positively contributed to the formation of non-essential amino acids [29], which is in line with our results, especially for serine, aspartic acid and glutamic acid (data not shown). In *Phaeodactylum tricornutum*, Jungandreas et al. [30] concluded that switching between red and blue light wavelengths, which caused vast metabolic reorganisation, was probably related to the engagement of different pathways for carbon partitioning [30]. *D. salina* DF15 cultivated under standard nitrogen level (1 g/L KNO_3_) delivered “high protein quality” with an essential amino acid index (EAAI) value of 0.99 under white light; when transferred to red or blue light for 24 h, the protein content was boosted, but interestingly, without a change in EAAI [11].

In the present study, biomass loss was evident by the end of the experiment under all light wavelengths (Figure 1). The biomass and protein contents achieved evidently depended on the strain, growth rate and light wavelength. Protein percentage was significantly higher for DF15 than for CCAP 19/30 across all wavelengths, but biomass accumulation was negatively affected by blue light compared to either red or white, in both strains. Certain amino acids were also reduced more than others (Figure 2B). Ultimately, across all wavelengths, the data indicated that the protein quality for both strains was less than ideal. In contrast, a “high protein quality” was achieved when DF15 was cultivated under similar conditions but with tenfold more nitrogen [11]. In *Synechocystis* sp. and *Isochrysis zhangjiangensis,* a brief period of N-starvation enhanced the production of several EAAs, but prolonged nitrogen starvation caused all EAA levels to drop sharply [25,27].

The EUC of *D. salina* also varied between two strains and across different light wavelengths. Nevertheless, even the highest EUC reached in *D. salina* DF15 under red light (3.2 g MSG/100 g AFDW) is still considerably lower than for other microalgae and foods such as meat and mushroom. The highest EUC found in *Rhodomonas salina* was reported to be 23.4 g MSG/100 g AFDW when cultivated at pH 8.5 and 30% salinity [31]. In Sous-vide beef, the EUC can rise as high as 53.3 g MSG/100 g AFDW [32]. In some savoury mushrooms, which are known for their umami flavour, the EUC ranges between 0.12 and 4465 g MSG/100 g AFDW in the fruit body and 1.92 and 460 g MSG/100 g AFDW in the mycelia [33]. Compared with these foods, *D. salina* appears to be low in umami flavour. In fact, some preliminary results have suggested that *D. salina* DF15 presents a floral smell and sweet taste, unlike the grassy and fishy taste that most other algae deliver (unpublished data). This offers an interesting path to further explore the taste profile of *D. salina* with sweetening effects.

### 4.2. Carotenoids Dynamics

The different effects of light wavelength on carotenoid production between the two strains is noteworthy. Carotenoids are essential for photosynthesis within microalgal chloroplasts where they are involved in both light harvesting and photo-protecting processes, and in stabilizing the structure of photosynthetic pigment–protein complexes and aiding in their function [1,2,3]. *D. salina* strains are known to accumulate up to 10% of the dry matter as β-carotene when the alga is exposed to high light intensities, or to the deprivation of nutrients or other growth-limiting conditions such as iron depletion, very high salt > 1.5 M, sub-optimal temperature and red light [4,5,6]; the amount depends on the integral irradiance per cell division cycle [6,34]. In the present study, CCAP 19/30 barely showed any carotenoid production, although maintained a high rate of biomass accumulation and delivered a high protein content, whereas DF 15 yielded more than 12% β-carotene AFDW across all wavelengths tested. Considering the ash content in *D. salina* to be around 20%, the value may correspond to just below 10% dry weight [35].

We previously reported that in *D. salina* CCAP 19/30, a decreased photosynthetic rate in light intensities of 200 and 500 µmol photons m^−2^ s^−1^ reduced the protein content due to photoinhibition, but at higher light intensities, intracellular glycerol increased and stabilized the photosynthetic apparatus. Under these conditions, the photosynthetic rate increased to a maximum level and delivered the highest protein content per cell. We have suggested that in *D. salina* CCAP 19/30, glycerol stabilizes the photosynthetic apparatus for maximum performance in high light intensities, a role normally attributed to carotenoids [36]. Under N-deprivation conditions imposed in the present study, accumulating glycerol instead of carotenoids in CCAP 19/30 may have been sufficient to sustain its biomass accumulation and deliver a high protein content measured as g/L; this warrants further investigation.

*D. salina* accumulates a high amount of β-carotene in the cell, of which, the *9-cis* isomer is more efficient in protecting against oxidative damage than its *all-trans* isomer. In this regard, *9-cis* β-carotene has greater market interest, e.g., in the pharmaceutical and cosmetic industries. Several studies have also reported the ratio of *9-cis* and *all-trans* β-carotene to be highly variable, depending on many factors such as light wavelengths, light intensity and temperature [5,14,36,37,38]. Red light not only increases the total carotenoids, but specifically the *9-cis* β-carotene content compared to white and blue light, reaching a *9-cis*/*all-trans* β-carotene ratio of around 1.2 under 1000 µmol photons m^−2^ s^−1^ red light for 24 h [5]. The effect of using red light was even more pronounced when combined with other factors such as lower temperature (15 °C) and light/dark cycle, resulting in *9-cis*/*all-trans* β-carotene ratios of more than 2 [5]. In the present study, a similarly high ratio of 1.5 was found under red light, much higher than those of white and blue light, and further confirms the effect of red light in enhancing *9-cis* β-carotene production in *D. salina* DF15. The over-production of *9-cis* β-carotene appears to trigger the expression of gene transcripts of β-carotene isomerase responsible for the catalytic conversion of *all-trans* to *9-cis* β-carotene under light stress conditions [5,39].

The appearance of phytoene in the biomass harvested after the cultivation of *D. salina* DF15 under different light wavelengths, especially blue light, is also noteworthy. Phytoene is the product of the first committed reaction of carotenogenesis, but a series of redox reactions normally convert it rapidly to carotenes. When phytoene desaturase (PDS) activity is inhibited by herbicides, phytoene accumulates, but its accumulation is normally enhanced under red light, not blue [6]. However, we recently showed that mitosis inhibitors such as chlorpropham may also support phytoene accumulation [6,40,41]; these appear to disrupt the synchronised control between nuclear and chloroplast events in *D. salina*, which affects the recruitment of carotenogenic enzymes into biologically active metabolons located in lipid membranes. Under nitrogen limitation, microalgal cells may not only redirect their metabolism to increase the degradation of certain amino acids and repress the biosynthesis pathways of others, but also redirect carbon and energy flows towards triacylglycerol (TAG) accumulation [22]. It is possible, therefore, that the production of functionally active lipid membranes normally required for an ordered series of redox reactions to convert phytoene to β-carotene had become impaired once nitrogen was depleted, especially under blue light.

## 5. Conclusions

The results from the present study show dissimilar responses of two *D. salina* strains cultivated under varying light wavelength. From analysis of the biochemical composition of the biomass harvested when nitrogen was depleted, protein percentages were higher for *D. salina* DF15 (27–31% AFDW) than for *D. salina* CCAP 19/30 (16–18% AFDW) across all wavelengths; however, in both strains, cultivation under blue light negatively affected biomass accumulation compared to under red or white light. *D. salina* DF15 produced more than 12% β-carotene over AFDW, with a very high *9-cis*/*all-trans* β-carotene ratio of 1.5 under red light. Phytoene production was also evident in *D. salina* DF15 under all conditions, and under blue light in particular (0.36% AFDW). In contrast, in *D. salina* CCAP19/30, carotenoid accumulation was negligible. The profile of EAA and calculation of the EAAI was less than ideal in terms of protein quality, for both strains: the highest EAAI values for *D. salina* DF15 and *D. salina* CCAP 19/30 were 0.8 under white light and 0.7 under blue light. *D. salina* DF15 had a higher umami concentration, whereas *D. salina* CCAP 19/30 resulted in a higher biomass concentration. The different effects appear to be related to the different photoprotective mechanisms operating in each strain, i.e., carotenoids in the case of *D. salina* DF15, but glycerol in *D. salina* CCAP.

## Figures and Tables

**Table 1:** Individual and total amino acid contents of *D. salina* DF15 and *D. salina* CCAP19/30 at the end of the experiment with the FAO/WHO reference.

mg/g Protein		Histidine	Isoleucine	Leucine	Lysine	Methionine + Cysteine	Phenylalanine + Tyrosine	Threonine	Valine	Total EAA
DF 15	White	12.1 ± 0.9	32.7 ± 0.8	43.9 ± 2.0	31.2 ± 2.6	13.1 ± 0.9	72.6 ± 4.5	19.6 ± 1.1	25.8 ± 0.8	251.0 ± 13.6 ^a^
	Red	9.1 ± 0.4	32.3 ± 0.5	42.5 ± 0.3	24.0 ± 0.4	13.6 ± 0.4	59.8 ± 0.4	21.0 ± 0.2	26.6 ± 0.2	228.9 ± 2.9 ^b^
	Blue	10.7 ± 0.8	34.1 ± 0.7	49.2 ± 0.2	33.1 ± 1.5	11.8 ± 0.2	68.1 ± 4.0	14.0 ± 0.3	25.3 ± 0.4	246.2 ± 8.1 ^ab^
CCAP 19/30	White	6.8 ± 0.5	24.5 ± 0.5	33.9 ± 0.2	20.5 ± 0.5	7.6 ± 0.2	43.2 ± 0.6	17.6 ± 0.1	19.5 ± 0.2	173.7 ± 2.7 ^c^
	Red	8.8 ± 0.6	28.1 ± 0.5	35.3 ± 0.3	21.9 ± 0.2	9.1 ± 0.4	48.3 ± 0.8	16.3 ± 0.5	21.2 ± 0.3	188.9 ± 3.6 ^c^
	Blue	10.4 ± 0.9	33.4 ± 0.6	43.2 ± 0.4	26.0 ± 0.6	11.0 ± 0.2	56.5 ± 0.8	23.2 ± 0.4	25.7 ± 0.4	229.5 ± 4.1 ^b^
FAO/WHO		15	30	59	45	22	38	23	39	271

Underlined values indicate levels above FAO/WHO reference. The different letters indicate statistically different total EAA (significance level at *p*-value < 0.05).

## Supplementary Materials

The following are available online at https://www.mdpi.com/article/10.3390/foods10112824/s1, Figure S1: Typical relative spectral power distribution of white, blue and red LED lights in the Algem^®^ bioreactor, Figure S2: Appearance of *D. salina* cultures at the end of each treatment. Cultivation was conducted at 25 °C, pH 7.5 and 100 rpm mixing using white, red and blue light at 200 µmol photons m^−2^ s^−1^., Figure S3: HPLC-DAD chromatograms of carotenoids extract from *D. salina* DF15 and *D. salina* CCAP 19/30 under different light wavelengths. The peaks are identified as: phytoene isomer 1 (1), phytoene isomer 2 (2), *all-trans* β-carotene (3) and *9-cis* β-carotene (4).
