# Supplementary material for: A Comparison of β-Carotene, Phytoene and Amino Acids Production in Dunaliella salina DF 15 (CCAP 19/41) and Dunaliella salina CCAP 19/30 Using Different Light Wavelengths
Source: Foods. 2021 Nov 16;10(11):2824. doi: 10.3390/foods10112824 (PMC8617983; doi:10.3390/foods10112824)
Supplement: Supplementary file 1 [file foods-10-02824-s001.zip › foods-1430273-supplementary.pdf]

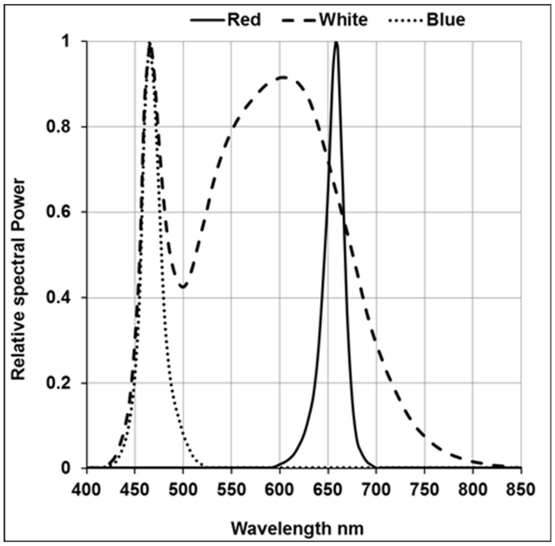

Figure S1. Typical relative spectral power distribution of white, blue and red LED lights in the Algem® bioreactor.

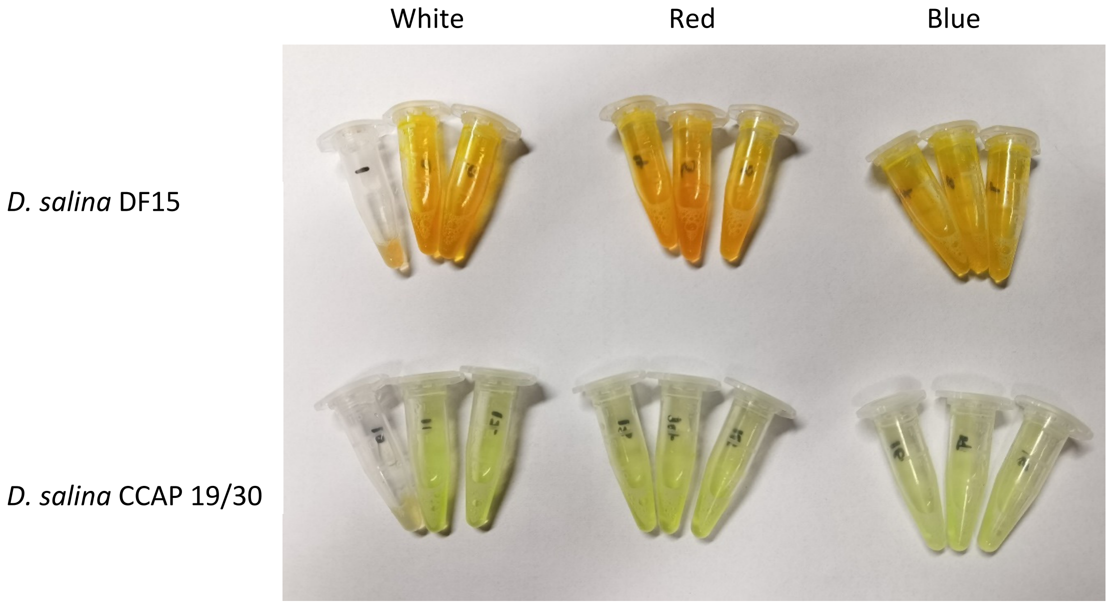

Figure S2: Appearance of *D. salina* cultures at the end of each treatment. Cultivation was conducted at 25°C, pH 7.5 and 100 rpm mixing using white, red and blue light at 200 µmol photons m^{-2} s^{-1}.

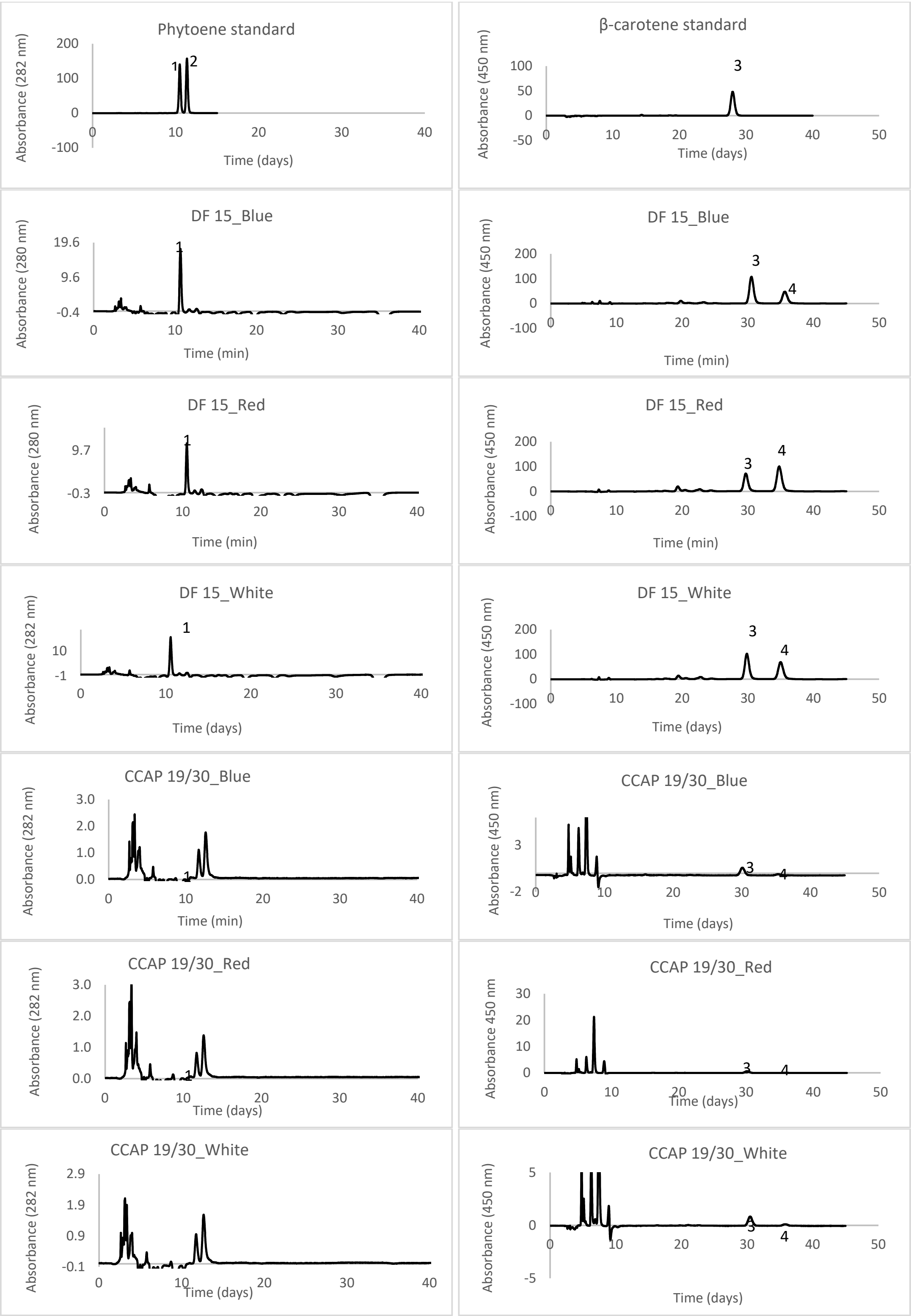

Figure S3: HPLC-DAD chromatograms of carotenoids extract from *D. salina* DF15 and *D. salina* CCAP 19/30 under different light wavelengths. The peaks are identified as: phytoene isomer 1 (1), phytoene isomer 2 (2), *all-trans* β-carotene (3) and *9-cis* β-carotene (4).
